# A case report and literature review of livedoid vasculopathy in children

**DOI:** 10.3389/fped.2025.1537133

**Published:** 2025-05-14

**Authors:** Jing Qu, Zhihong Hao, Wang Junli, Shengyou Yu

**Affiliations:** ^1^ Department of Pediatrics, Guangzhou First People’s Hospital, Guangdong Medical University, Zhanjiang, Guangdong, China; ^2^ Department of Pediatrics, Guangzhou First People’s Hospital, School of Medicine, South China University of Technology, Guangzhou, Guangdong, China; ^3^ Department of Pediatrics, The Second Affiliated Hospital, South China University of Technology, Guangzhou, China

**Keywords:** livedoid vasculopathy (LV), white atrophy, rivaroxaban, capillaries, children

## Abstract

**Background:**

Livedoid vasculopathy (LV) is a rare, non-inflammatory, intradermal vascular obstructive skin disorder characterized by purpuric papules and plaques with capillary dilation. These lesions typically progress to crusted ulcers and ultimately result in fixed, white, atrophic stellate scars. The condition is marked by painful ulcers that heal slowly and have a tendency to recur.

**Case presentation:**

We report a case of a pediatric patient presenting with recurrent rashes and pain in both lower extremities. Physical examination revealed purpuric plaques with ulceration, scarring, and white atrophic healing features. Histopathological examination demonstrated intradermal thrombosis, vessel wall necrosis, and surrounding inflammatory cell infiltration with erythrocyte extravasation. Periodic acid-Schiff (PAS) staining was positive. The clinical and pathological findings were consistent with a diagnosis of LV. The patient was treated with oral rivaroxaban.

**Conclusion:**

This case highlights the critical importance of early recognition and intervention in the management of LV. Clinicians should consider LV in the differential diagnosis when encountering patients with painful purpuric rashes. Improvement in pain following treatment with anticoagulants, such as rivaroxaban, may indirectly support the diagnosis. A skin biopsy is essential for definitive diagnosis.

## Introduction

Livedoid vasculopathy (LV) is a rare, non-inflammatory, intradermal vascular obstructive skin disease characterized by painful ulcers, reticular cyanotic macules, and porcelain-white atrophic scarring, predominantly affecting the distal portions of the lower extremities (1–3). Although LV was first described in the early 20th century, it was not until the revision of the International Classification of Diseases (ICD-10) in 2004 that the condition was accurately defined. LV is a chronic, relapsing disorder with a distinct seasonal pattern, typically exacerbating in the summer and remitting in the winter (4). The annual incidence of LV is approximately one case per 100,000 individuals, with a predilection for females (5). Despite its clinical significance, the international diagnostic criteria for LV remain poorly defined, with only an expert consensus published in 2013 providing guidance (6). The non-specific clinical manifestations of LV often necessitate histopathological confirmation via skin biopsy for accurate diagnosis. The most typical pathological feature of LV is focal thrombosis within the dermal capillaries (3).

Given the unique clinical and pathological characteristics of LV, differentiating it from other vasculitic diseases is crucial, making clinical diagnosis particularly challenging. Case reports of LV play a vital role in guiding clinical practice, providing valuable insights for the diagnosis and treatment of this condition. They also contribute to the development of more rational diagnostic and therapeutic strategies for similar patients.

## Case presentation

### Description of patient

A 13-year-old male patient from Guangdong, China, was admitted to our hospital on 23 September 2023, with a 15-day history of recurrent rash and pain in both lower extremities. The patient had been previously diagnosed with “Henoch–Schönlein purpura (HSP)” in January 2021 due to a generalized red rash. The rash began from the lower limbs and spread to the whole body, which resolved with oral cimetidine and cetirizine. He was discharged for regular checkups, during which he received methotrexate 7.5 mg once a week without any recurrence of the disease. In June 2023, the rash reappeared. The local hospital diagnosed the recurrence of allergic purpura, and the symptoms were relieved after 2 weeks of symptomatic drugs—cimetidine and methylprednisolone.

**Figure 1:**
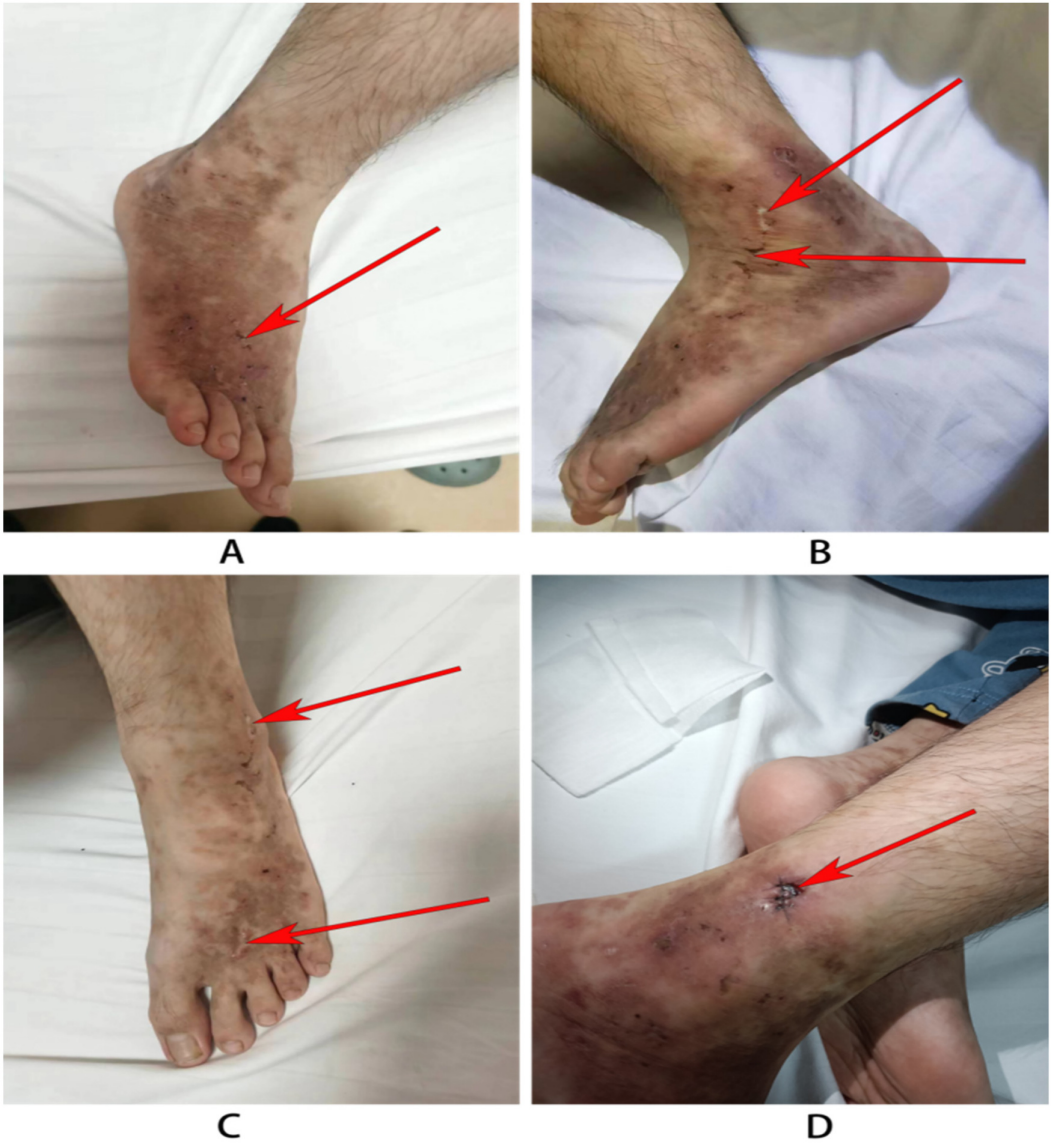
Skin rash in the child. **(A)** The left leg of the child: atrophic scarring around lateral malleoli (the red arrow). **(B,C)** The right legs: The arrow indicates porcelain-white atrophic scarring and stellate purpuric macules with telangiectasias. **(D)** Wound recovery after 3 days after dermatological pathology.

On admission, scattered old rashes were noted throughout the body, with new red eruptions on both lower limbs that did not fade with pressure, accompanied by breakouts and crusts, without pruritus (Figure 1). Hypopigmented spots with a white atrophic appearance were also observed. Ultrasound examination of the extremities performed at an outside hospital did not reveal any abnormalities in the arteries and veins of both lower extremities.

### Clinical findings and diagnostic assessment

Upon admission to our hospital, a comprehensive set of laboratory tests (Table 1) was performed, including assessments of coagulation factors, D-dimer levels, vasculitis markers, humoral immunity, pre-transfusion screening, autoantibody profiles, and others. These tests did not reveal any significant abnormalities, effectively ruling out the possibility of HSP recurrence.

**Table 1:** Laboratory data at the admission of the patient.

Item	Data	Experimental test method
Routine blood test		
WBC	3.88 × 10 × 10^9^/L	Flow cytometry
RBC	4.48 × 10 × 10^12^/L	Resistance method
HGB	134.00 g/L	SLS–hemoglobin test
PLT	209.00 × 10 × 10^9^/L	Resistance/optical method
Neut#	1.57 × 10 × 10^9^/L	Computation
Lymph#	1.79 × 10 × 10^9^/L	Computation
Coagulation function		
TT	16.2 s	Magnetic bead method
PT_SEC	14.2 s	Magnetic bead method
PT_INR	1.07 INR	Computation
PT%	88%	Computation
APTT	37.9 s	Magnetic bead method
APTT_R	1.11	Computation
FIB	2.84 g/L	Magnetic bead method
D-dimer (D-D)	410 g/L	Immunoturbidimetry
Immunity		
MPO IgG	2.69 AU/ml	Chemiluminescence
PR3 IgG	<2.00 AU/ml	Chemiluminescence
ACA IgG	2.75 GPLU/ml	Chemiluminescence
ACA IgM	<2.00 MPLU/ml	Chemiluminescence
ds-DNA	–	Immunoblotting
rRNP	–	Immunoblotting
ssA	–	Immunoblotting
ssB	–	Immunoblotting
Sm	–	Immunoblotting
nRNP	–	Immunoblotting
Jo-1	–	Immunoblotting
Scl-70	–	Immunoblotting
AauA	–	Immunoblotting
CENP B	–	Immunoblotting
Histone	–	Immunoblotting
ANA	4.68 AU/ml	Chemiluminescence
IgA	1.3000 g/L	Scattering ratio turbidimetry
IgG	10.800 g/L	Scattering ratio turbidimetry
IgM	1.6800 g/L	Scattering ratio turbidimetry
IgE	19.630 ng/ml	Electrochemiluminescence

**Figure 2:**
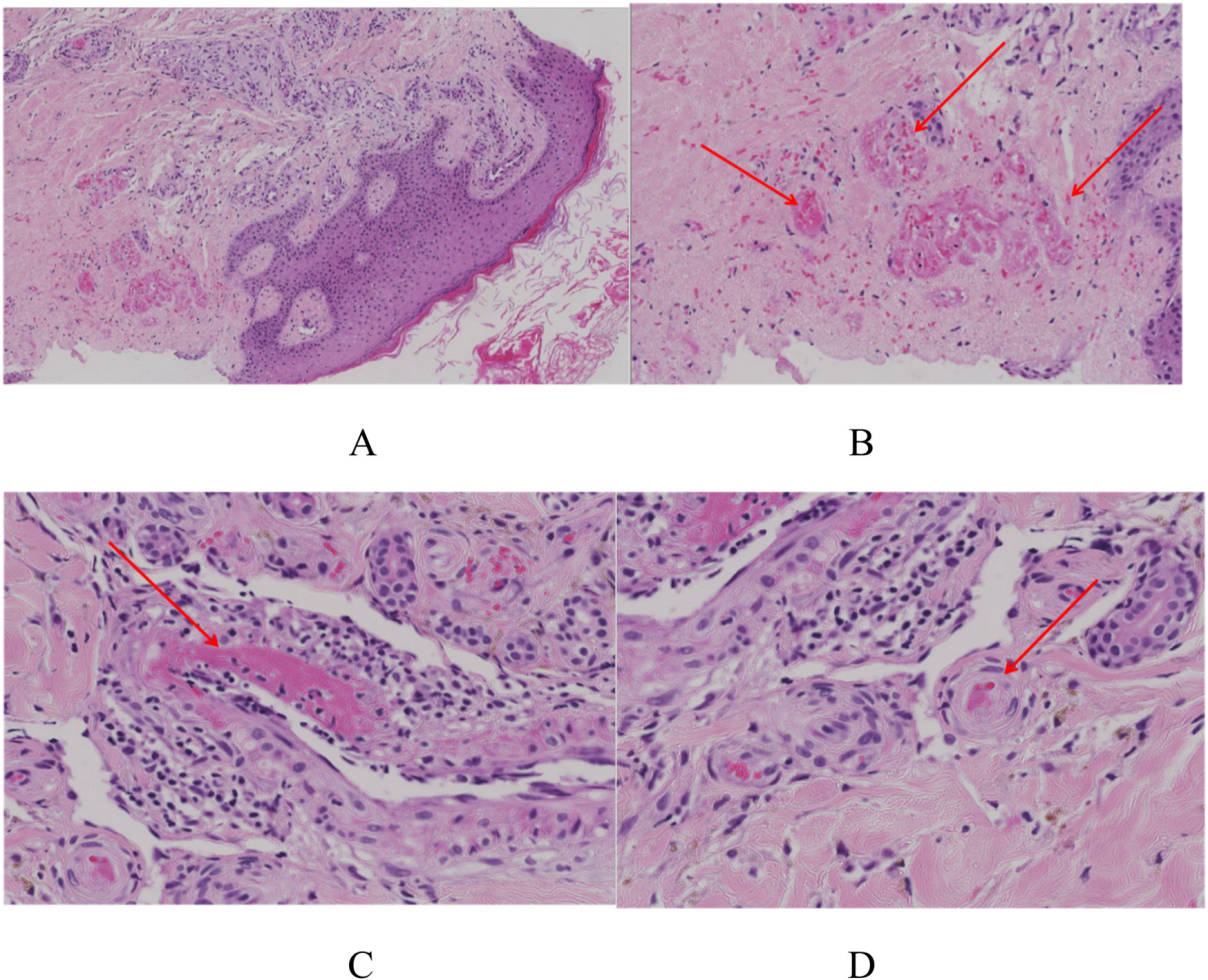
The skin biopsy of this child. **(A)** The overall picture of skin HE staining. H&E 8×. **(B)** Capillary telangiectasia, extravasation of red blood cells, and intraluminal capillary thrombi (the red arrows). H&E 10×. **(C)** Livedoid vasculopathy in a skin biopsy with an occlusive, intraluminal thrombus (the red arrows) surrounding hemorrhage. H&E 40×. **(D)** Chronic change: thickening of capillary walls with pink, glassy (hyalinized) collagen (the red arrows). H&E 40×.

**Figure 3:**
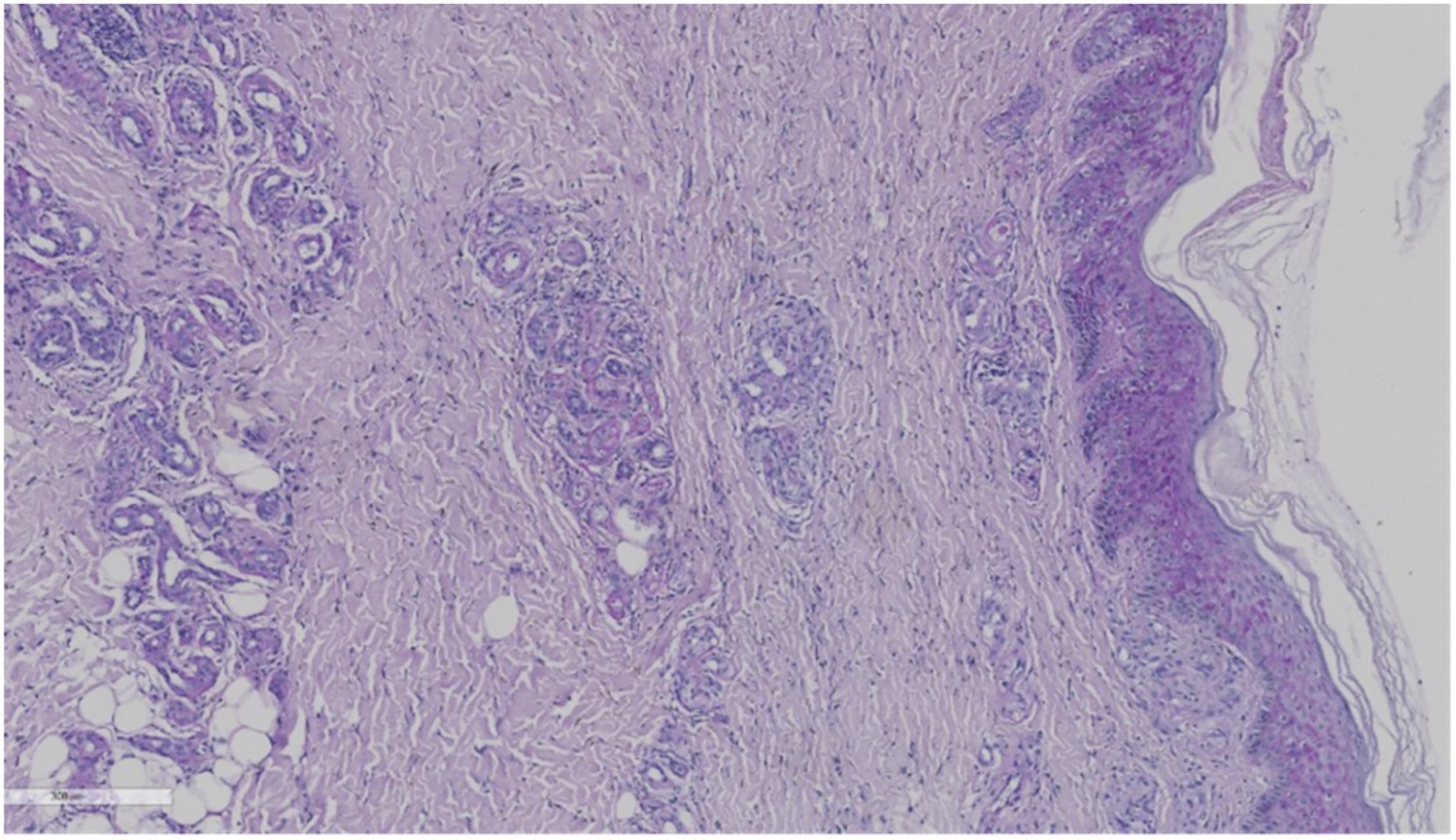
Immunofluorescence examination showing that IgG, C3, IgM, and IgA are negative.

The skin biopsy was requested on September 26. A skin specimen measuring approximately 1 cm × 0.6 cm × 0.5 cm was taken from the right ankle for pathological examination. Microscopic examination of the specimen revealed normal-appearing squamous epithelium on the skin surface, with hyperplasia of small blood vessels in both the superficial and deep dermis, accompanied by a small number of inflammatory cells. No tumor cells were identified. Direct immunofluorescence testing showed negative results for IgG, C3, IgM, and IgA (Figures 2, 3).

After a thorough evaluation of the patient's condition, the skin specimen was reviewed twice in consultation with dermatology. Histopathological examination revealed epidermal hyperkeratosis, intravascular thrombosis, and necrosis of the vessel wall in the dermis, along with perivascular inflammatory cell infiltration and erythrocyte extravasation. Special staining techniques, such as periodic acid-Schiff (PAS) staining, were positive, further confirming the diagnosis of livedoid vasculopathy (LV) (Figure 3).

The patient, a male adolescent, presented with “rash and pain” as the primary symptoms. He had previously been diagnosed with HSP, and his symptoms had improved with treatment. On admission, physical examination revealed new erythematous eruptions on both lower limbs that did not blanch with pressure, accompanied by breakouts and crusts but no pruritus. Laboratory tests evaluated immune antibodies and coagulation function, ruling out HSP and other immune-mediated diseases. Skin biopsy findings suggested non-inflammatory vascular wall damage and fibrinoid necrosis of the vessel wall. Based on these findings, the patient was ultimately diagnosed with LV.

### Therapeutic intervention

Following the diagnosis of LV, the patient was initiated on anticoagulation therapy with intravenous heparin and oral rivaroxaban (10 mg once daily). One week later, the patient's pain was significantly relieved, and he was discharged without recurrence of the rash.

### Follow-up and outcomes

The patient continued to take rivaroxaban regularly for 2 months. Follow-up visits revealed a significant reduction in the rash compared with the initial presentation, with no new eruptions observed. The patient was discharged with regular follow-up appointments, and his condition showed marked improvement compared with the initial presentation.

## Discussion

LV has a strong gender characteristic; the incidence of women is three times that of men, especially in the age group of 15–50 years old (7). As a disease related to coagulation disorders, it is divided into secondary and primary according to the presence or absence of primary disease. It is often associated with multiple stasis, autoimmune connective tissue diseases, tumors, and immune-related diseases (6). The disease is often exacerbated by temperature changes and a hypercoagulable state of the blood (2, 8). Therefore, clinicians must conduct a comprehensive assessment to diagnose LV accurately and manage its underlying causes. In our case, the patient had a history of Henoch–Schönlein purpura (HSP), which significantly increased the risk of vascular fragility and endothelial damage due to an autoimmune attack. This predisposition to vascular endothelial damage further heightened the likelihood of thrombosis. Upon admission, we first evaluated and ruled out the recurrence of HSP based on clinical and laboratory findings.

The pathogenesis of LV remains elusive, with abnormal coagulation function being the most likely underlying mechanism. Current research primarily focuses on three aspects: flow disruption, endothelial injury, and coagulation disorders (3). This patient had suffered from the basic disease of allergic purpura, which greatly increased the possibility of vascular fragility and endothelial damage after his own immune attack. It has a high-risk factor for vascular endothelial damage, making thrombosis even easier. In pathology, the three major manifestations of LV disease are segmental hyalinization or fibrinoid degeneration of dermal vessels, proliferation of the endothelium, and intraluminal thrombosis (3, 7). The skin biopsy of this patient showed capillary telangiectasia, extravasation of red blood cells, and intraluminal capillary thrombi. Clinically, the rash presented as erythematous macules that were not significantly elevated above the skin surface and were devoid of itching or pain. The lesions progressed through various stages, including ulceration, white atrophic-like changes, and crusting. LV predominantly affects the lower limbs, with rare involvement of the upper limbs (7). Capillary embolism leads to the formation of small ulcers, which typically develop into characteristic “atrophie blanche” 3–4 months later. This condition is often accompanied by burning pain, significantly impacting the patient's quality of life, particularly during active disease phases, affecting psychological, physical, and social aspects (9).

In terms of clinical laboratory findings, most cases of LV lack significant diagnostic markers. However, some studies have suggested an association with hypercoagulable states. For instance, a prospective study found elevated levels of hypercoagulability markers, such as fibrinopeptide A, lipoprotein (a), and plasminogen activator inhibitor-1 (10). In our case, only routine laboratory tests were performed, and more specialized studies were lacking. Currently, various therapeutic regimens are employed for LV, yet standardized treatment protocols remain limited. Among monotherapy options, anticoagulants are the most commonly used, followed by systemic glucocorticoids, antiplatelet agents, and immunomodulatory drugs (2, 11). Given the low incidence of LV, research on its treatment is relatively sparse. First-line treatments typically include antiplatelet drugs, such as aspirin and clopidogrel. Rivaroxaban has emerged as a promising therapeutic option due to its safety and ease of use. A comparative study analyzed 20 articles and 138 patients, finding that the average treatment response time was 2.4 months for rivaroxaban vs. 2.3 months for intravenous immunoglobulin (IVIG). This study further validated the efficacy of rivaroxaban (12).

Rivaroxaban is a direct Xa factor inhibitor that has been widely used to treat and prevent major thromboembolic diseases. Compared with other anticoagulants such as low molecular weight heparin and warfarin, rivaroxaban can be taken orally and does not require international normalized monitoring, making it the preferred drug. A study by Kerk et al. (13) first reported a successful case of treating LV with rivaroxaban. Rivaroxaban was gradually becoming familiar to people. In a recent review, approximately 73 patients were counted, and the therapeutic dose of lifasab was 10–20 mg/day. Approximately 82.2% (60/73) responded to the treatment, and their pain and ulcers were relieved (14). A cross-sectional study by Zhao et al. (15) found that it can monitor the active phase through coagulation factor X (IQR: 102.3–132.5 vs. IQR: 92.9–118.8, *P* = 0.04). Moreover, 73% of patients achieved complete remission within 12 weeks of rivaroxaban treatment, with low side effects (25%). It is clear from these studies that rivaroxaban is effective for LV patients. In our case, it also demonstrated that appropriate rivaroxaban use in children can effectively control the progression of LV disease. Adverse reactions were observed rarely, with heavy menstrual bleeding being the most common (13, 14).

Rivaroxaban is a direct factor Xa inhibitor widely used for treating and preventing major thromboembolic diseases. Compared to other anticoagulants, such as low molecular weight heparin and warfarin, rivaroxaban offers the advantage of oral administration without the need for international normalized ratio (INR) monitoring, making it a preferred choice (13). A recent review summarized data from 73 patients, with a therapeutic dose of rivaroxaban ranging from 10 to 20 mg/day. Approximately 82.2% (60/73) of patients responded to treatment, experiencing relief from pain and ulceration (14). A cross-sectional study demonstrated that coagulation factor X levels could be used to monitor disease activity. Moreover, 73% of patients achieved complete remission within 12 weeks of rivaroxaban treatment, with a low incidence of adverse effects (14). These studies collectively highlight the efficacy of rivaroxaban in treating LV. In our case, rivaroxaban effectively controlled the progression of LV in the pediatric patient, with minimal adverse reactions, the most common being heavy menstrual bleeding (13, 14). In addition to rivaroxaban, emerging targeted therapies show promise in treating refractory LV. For instance, tofacitinib, a pan-Janus kinase (JAK) inhibitor, has been reported to be effective in LV treatment (16). Another study demonstrated that anti-interleukin 17 A biologics can also control disease progression (17). In conclusion, the application of targeted therapies has expanded our understanding of the therapeutic mechanisms of LV, bringing the possibility of clinical cure closer to reality.

## Conclusion

When patients have a purple rash with pain, clinicians should consider the possibility of LV. It is important to first assess whether the child has a hypercoagulable state before analyzing the test results. There is no specific clinical test for this disease, and its diagnosis mainly depends on skin biopsy. However, invasive clinical examinations are difficult for family members to accept and increase the complexity of diagnosing and treating the disease. When diagnosis is challenging, anticoagulants such as rivaroxaban for treatment may be considered. If pain improves, it can indirectly confirm the condition. A skin biopsy must focus on dermal vascular occlusion, which is crucial for diagnosis.

## Data Availability

The original contributions presented in the study are included in the article/Supplementary Material, further inquiries can be directed to the corresponding author.
